# Supplementary material for: Broadly neutralizing nanobodies target a defined structural pivot site on the RSV fusion protein
Source: EMBO Mol Med. 2026 Apr 7;18(5):1866–83. doi: 10.1038/s44321-026-00412-w (PMC13179361; doi:10.1038/s44321-026-00412-w)
Supplement: Supplementary file 1 — Appendix [file 44321_2026_412_MOESM1_ESM.pdf]

Appendix

Appendix Figure S1. RSV F antigen preparation and Dromedary immunization. (Page 2)

Appendix Figure S2. Construction of a phage display library. (Page 3)

Appendix Figure S3. Immunofluorescence results of candidate Nb-Fc fusions neutralizing RSV A2 virus in vitro. (Page 4)

Appendix Figure S4. In vitro RSV A2 neutralization of the candidate Nb-Fc fusions. (Page 5)

Appendix Figure S5. Binding activity of candidate Nb-Fc fusions was analyzed by immunofluorescence. (Page 6)

Appendix Figure S6. The neutralizing activity of nanobodies 1G9 and 1D8 against RSV A2, RSV B and RSV Long subtypes. (Page 7)

Appendix Figure S7. The fusion inhibition of nanobodies 1G9 and 1D8 against RSV A2, RSV B and RSV Long subtypes. (Page 8)

Appendix Figure S8. Single-particle cryo-EM image processing workflow and global and local resolution estimations for the RSV A2 DS-Cav1:1G9 complex. (Page 9)

Appendix Figure S9. Single-particle cryo-EM image processing workflow and global and local resolution estimations for the RSV A2 DS-Cav1:1D8 complex. (Page 10)

Appendix Figure S10. The comparison of the epitopes of 1G9 (A) and 1D8 (B) with those of six previously reported antibodies. (Page 11)

Appendix Figure S11. Epitope comparison of 1G9 and 1D8 with three other antibodies targeting antigenic site IV. (Page 12)

Appendix Figure S12. The sequencing and alignment analysis of Nbs1G9 and 1D8. (Page 13)

Appendix Figure S13. Genetic sequencing and alignment analysis of F gene in RSVB [CH93(18)-18] and RSV B9320. (Page 14)

Appendix Table S1. Cryo-EM data collection, refinement and validation. (Page 15)

Appendix Table S2. Binding site of nanobodies on RSV F and sequence conservation. (Page 16)

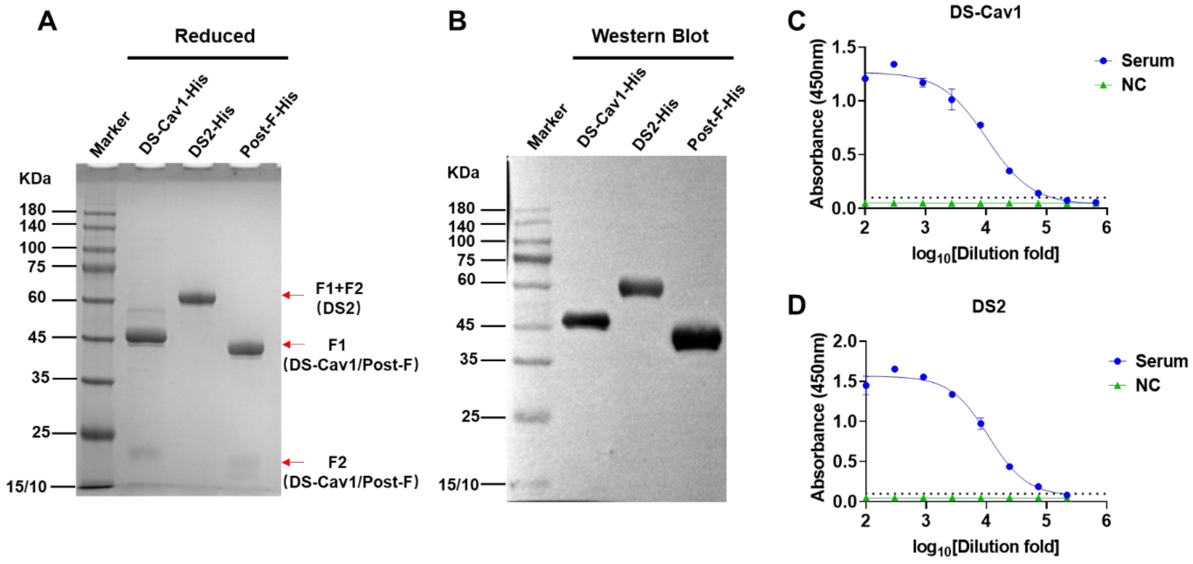

Appendix Figure S1. RSV F antigen preparation and Dromedary immunization. (A) Reduced SDS-PAGE analysis of the purified RSV pre-F (DS-Cav1 and DS2) and Post-F. The monomeric forms of DS1, DS2, and Post-F antigens showed molecular weights of 40-60 kDa. (B) Western Blot identification of purified DS-Cav1, DS2 and Post-F proteins using the Palivizumab as the primary antibody. (C-D) Following immunization dromedary, serum samples were collected to assess RSV pre-F-specific binding antibody titers by ELISA. NC, pre-immune serum. Data are average values of two replicates.

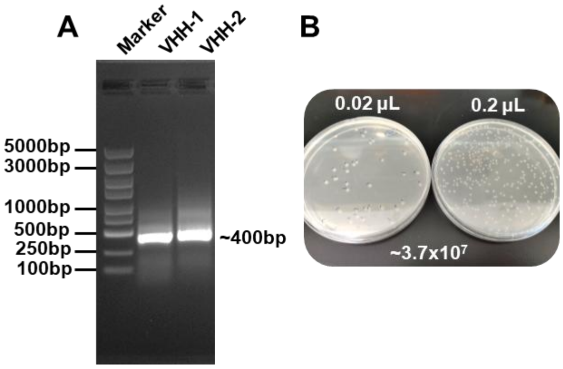

C

Name	RSV F (DS-Cav1/DS2)	
Library size	3.7x10^7	
Output phage of 2nd round	5.6*10^5 (DS-Cav1)	3.6*10^5 (DS2)
Monoclonal phage	96	96
Positive monoclonal phage	95	94
Different Sequence	75	75
Expressed proteins	55	

Appendix Figure S2. Construction of a phage display library. (A) Acquisition of VHH coding regions from immunized dromedary PBMCs through PCR. (B) The calculation result for phage library capacity. (C) Enrichment of phages after two rounds of panning on DS-Cav1 and DS2. The number of positive monoclonal clones (OD_{450}>0.5) and comparison of sequence diversity.

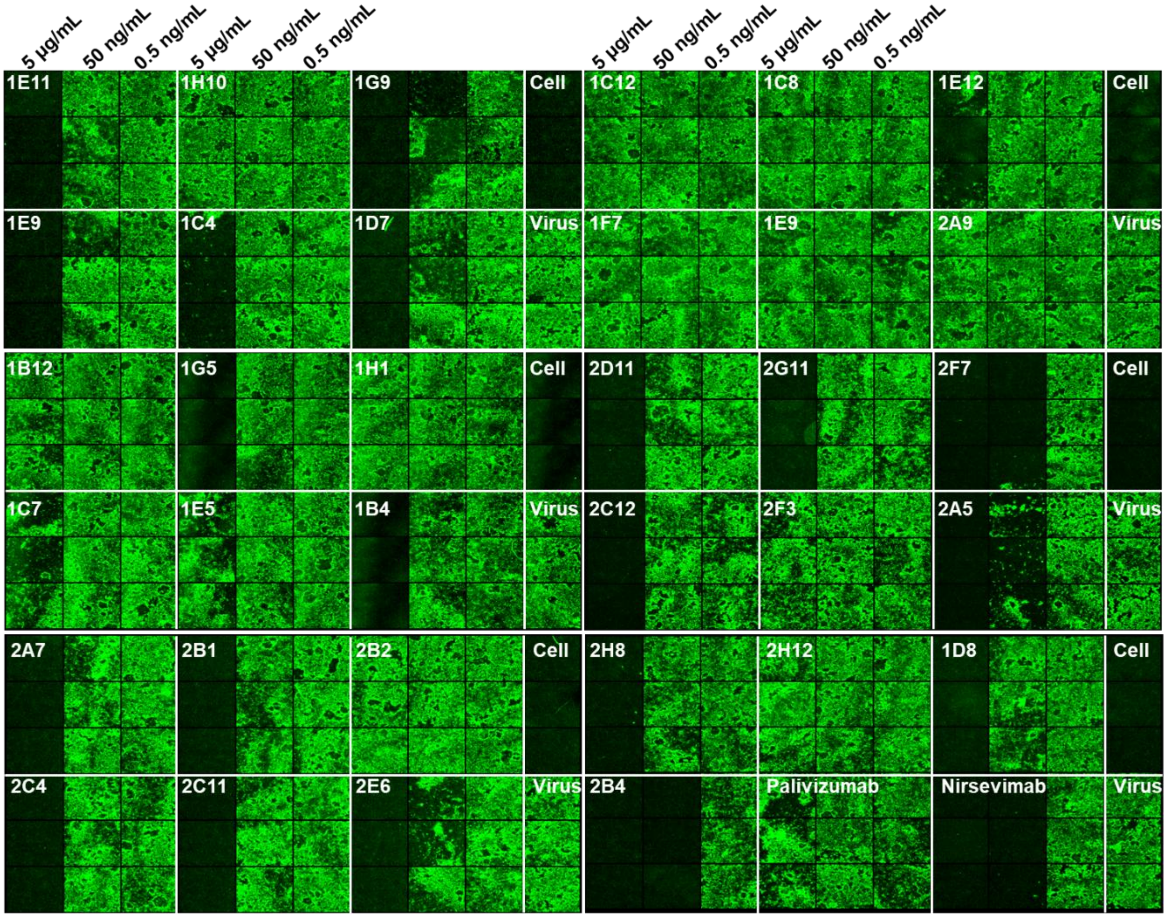

Appendix Figure S3. Immunofluorescence results of candidate Nb-Fc fusions neutralizing RSV A2 virus in vitro. Screening results for the neutralizing capacity of 34 nanobodies. White text in the upper left corner indicates the nanobody number. "Cell" represents the blank control group (no virus, no antibody). "Virus" represents the virus-only control group (virus added, no antibody). RSV F protein marker detected by antibody F-E2, the concentration of F-E2 used for detection is 1 μg/mL. Magnification: 10×. Palivizumab and nirsevimab were used as positive control neutralizing antibodies.

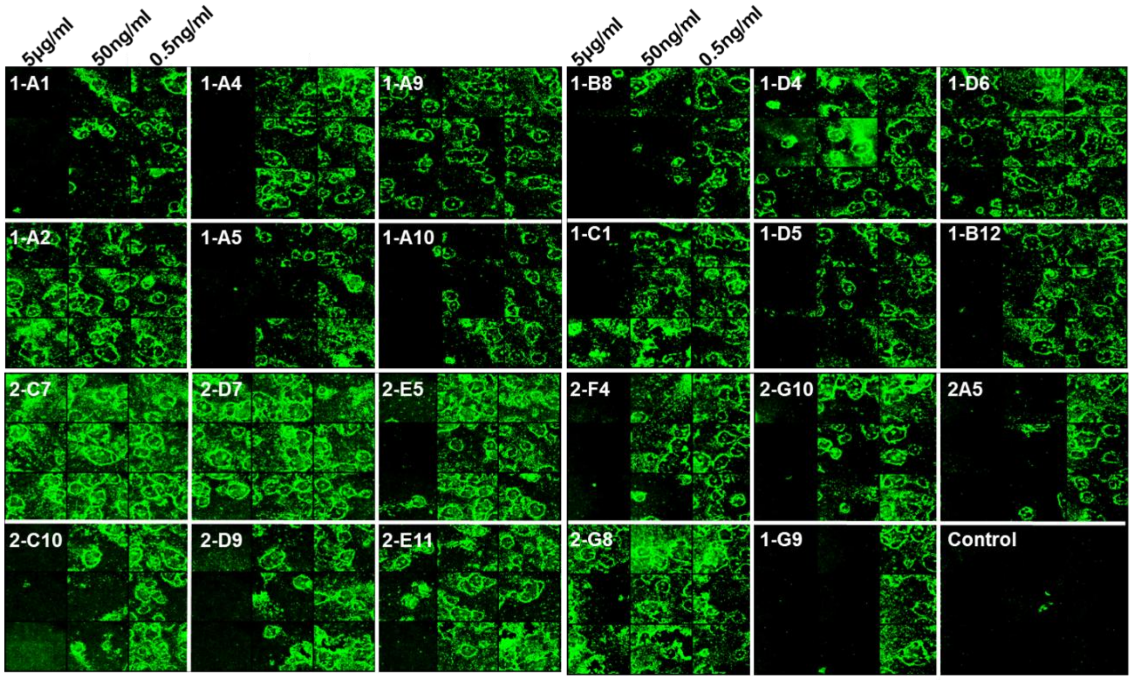

Appendix Figure S4. In vitro RSV A2 neutralization of the candidate Nb-Fc fusions. Screening results for the neutralizing capacity of 21 nanobodies. White text in the upper left corner indicates the nanobody number. "Control" represents the blank control group (no virus, no antibody). RSV F protein marker detected by antibody F-E2. the concentration of F-E2 used for detection is 1 μg/mL. Magnification: 10×.

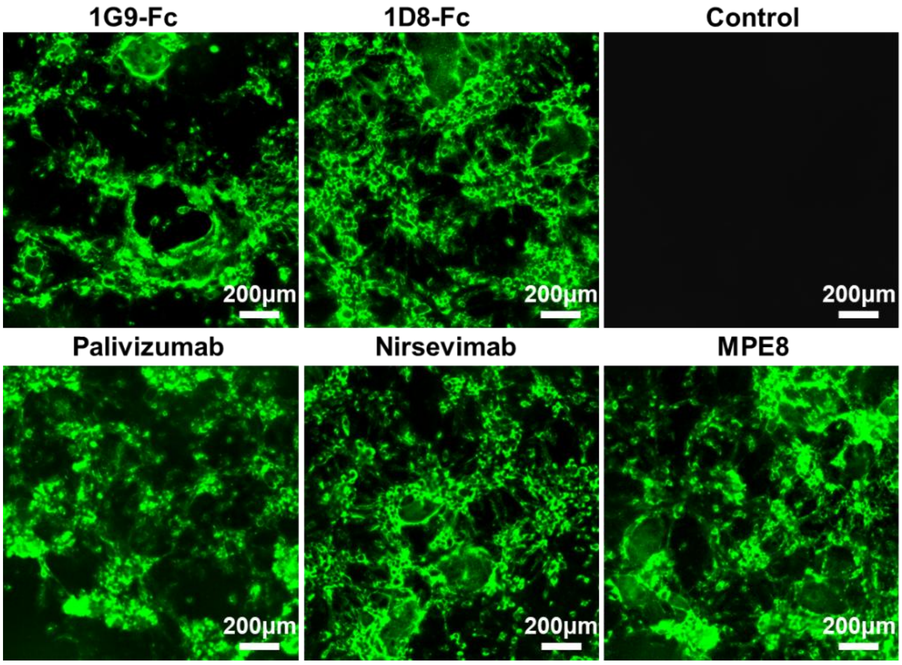

Appendix Figure S5. Binding activity of candidate Nb-Fc fusions was analyzed by immunofluorescence. Binding of the purified Nb-Fc fusions (1G9-Fc and 1D8-Fc) to native F protein (RSV A2) was detected by indirect immunofluorescence assay. Palivizumab, nirsevimab and MPE8 were used as positive control. The concentration of nanobody/antibody used for detection is 10 μg/mL. Scale bar: 200 μm. "Control" represents the blank control group (no virus, no antibody).

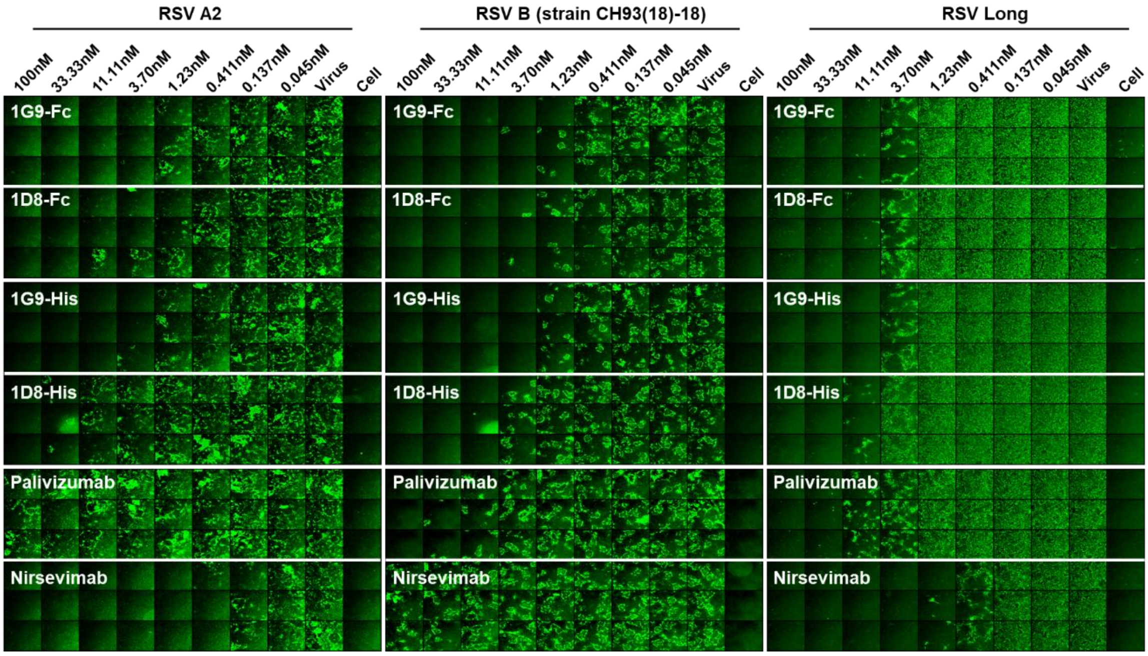

Appendix Figure S6. The neutralizing activity of nanobodies 1G9 and 1D8 against RSV A2, RSV B and RSV Long subtypes. The nanobodies were serially diluted and incubated with RSV for 1 h at 37°C. Then, mixtures were added to 96 plates and incubated for 2 h at 37°C. After changing fresh DMEM containing 2% FBS and 2% PS, Vero cells are grown at 37°C. After 72 h, cells were fixed, and RSV F was stained with primary antibody (F-E2, 1 μg/mL) and FITC-anti-human Fc (green, 1:2000). "Virus" represents the virus-only control group (virus added, no antibody). "Cell" represents the blank control group (no virus, no antibody).

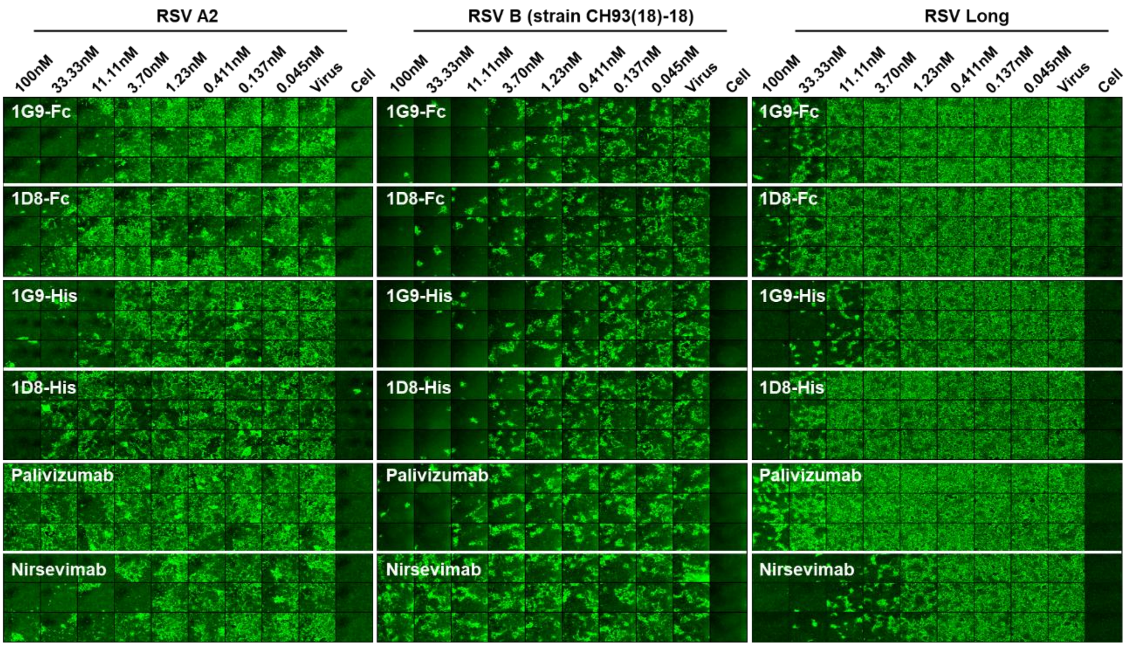

Appendix Figure S7. The fusion inhibition of nanobodies 1G9 and 1D8 against RSV A2, RSV B and RSV Long subtypes. (B) RSV A2, RSV B and RSV Long were pre-incubated in Vero cells for 1 h at 4°C to allow virus attachment. The nanobodies 1G9 and 1D8 were serially diluted in DMEM (2% FBS) and added to plates, and continuously incubated at 4°C. Finally, Vero cells were cultured at 37°C for 72 h. RSV F was stained with primary antibody (F-E2, 1 μg/mL) and FITC-anti-human Fc (green, 1:2000). "Virus" represents the virus-only control group (virus added, no antibody). "Cell" represents the blank control group (no virus, no antibody).

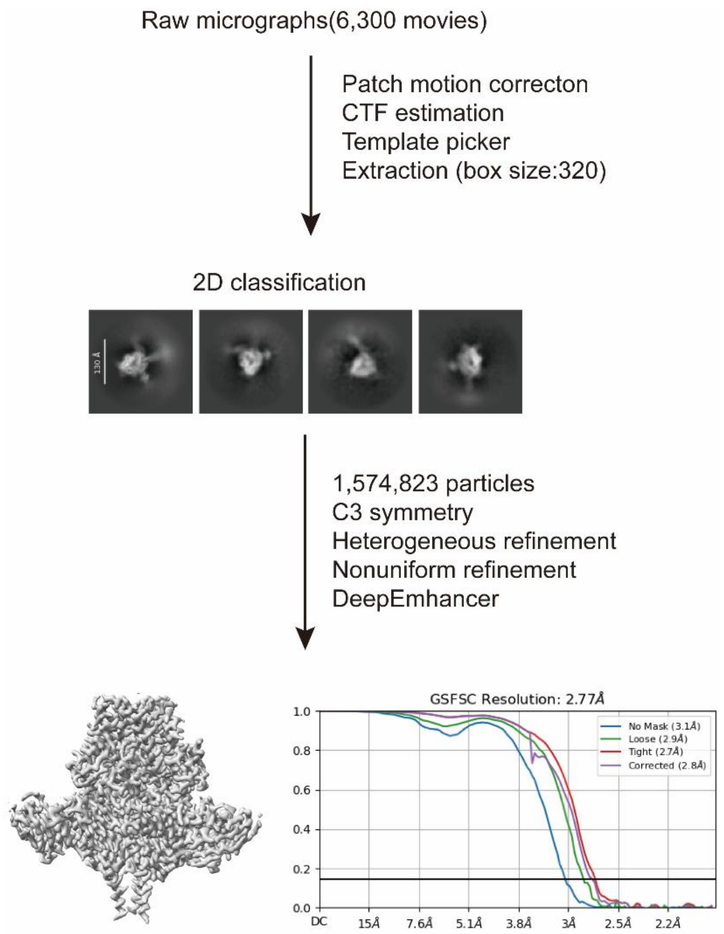

Appendix Figure S8. Single-particle cryo-EM image processing workflow and global and local resolution estimations for the RSV A2 DS-Cav1:1G9 complex. Representative 2D classification results, maps after DeepEmhancer sharpening, FSC curves are shown.

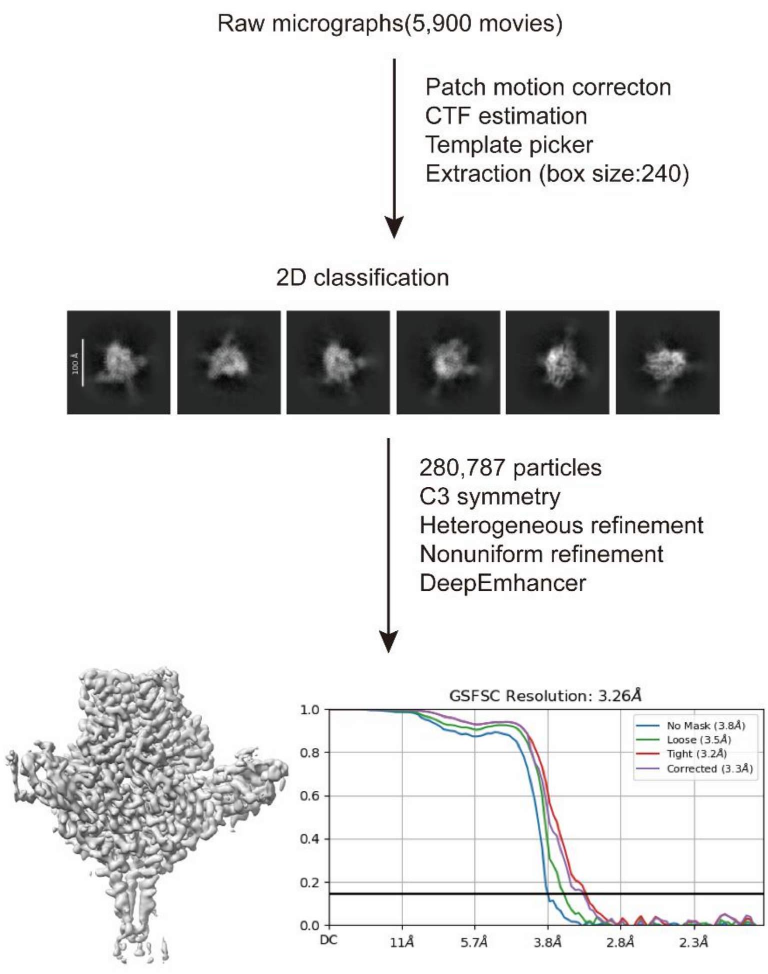

Appendix Figure S9. Single-particle cryo-EM image processing workflow and global and local resolution estimations for the RSV A2 DS-Cav1:1D8 complex. Representative 2D classification results, maps after DeepEmhancer sharpening, FSC curves are shown.

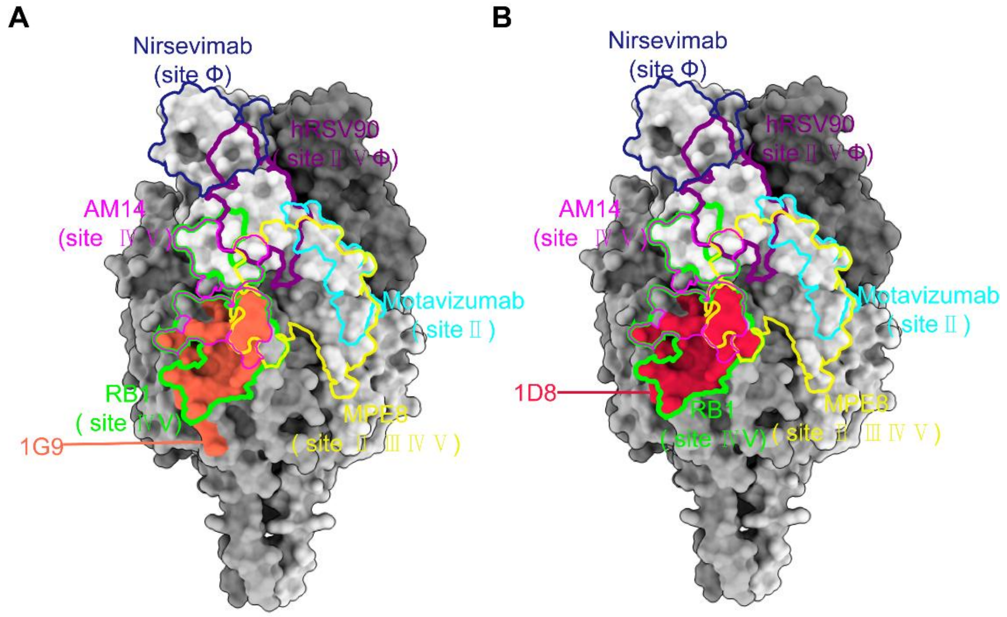

Appendix Figure S10. The comparison of the epitopes of 1G9 (A) and 1D8 (B) with those of six previously reported antibodies. Nirsevimab (site Ø, blue line), AM14 (magenta line), motavizumab (site II, cyan line), RB1 (site IV and V, lime line), hRSV90 (sites II, V, and Ø, purple line), and MPE8 (sites II, III, IV, and V, yellow line). The protomers of the F protein are colored light gray, gray, and dark gray, respectively. The epitopes of the nanobodies are highlighted in the same color as the nanobody they represent.

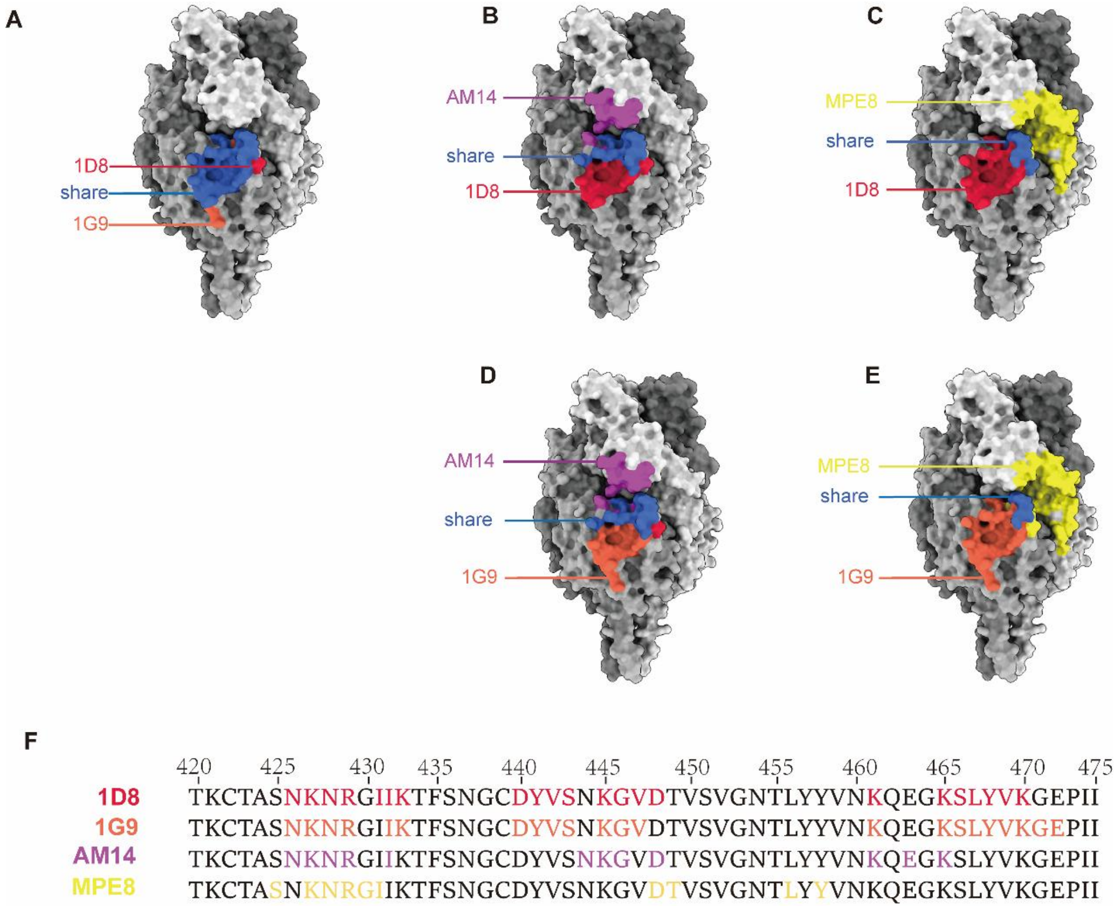

Appendix Figure S11. Epitope comparison of 1G9 and 1D8 with three other antibodies targeting antigenic site IV. (A-C) Epitope comparison of 1D8 (red) with 1G9 (orange), AM14 (purple, PDB: 7MMN), and MPE8 (yellow, PDB: 5U68). Residues shared across the interfaces are highlighted in blue. (D-E) Epitope comparison of 1G9 (orange) with AM14 (purple, PDB: 7MMN) and MPE8 (yellow, PDB: 5U68). Residues shared across the interfaces are highlighted in blue. (F) The interface of 1D8 (crimson), 1G9 (orange), AM14 (purple), and MPE8 (yellow) on RSV-F was analyzed using PDBePISA. Residues located within the structural pivot region are colored as described above.

```
           1        10        20        30        40        50        60        70        80        90        100       110       120     128
           |--------+---------+---------+---------+---------+---------+---------+---------+---------+---------+---------+---------+-------|
1G9        QLQLVESGGGAVQSGGSLRLSCAASGDTYSIYSMGWFRQAPGREREGVAAIFSDGSTNYAASVEGRFTISQDNAENTLYLQMNSLKPEDTAMYFCAAG--NAAWGLVDYYTEADFAYWGQGTQVTVSS
1D8        QLQLVESGGGLVQAGGSLRLSCAASEHTYCFYDMSWYRQAPGKEREFVSGIDIDGSARYADSVKGRFTISQDNAANTVYLQMNSLKPEDTAMYYCKTRLCGEADTYCSGGDCRDSGYWGQGTQVTVSS
Consensus  QLQLVESGGGAVQAGGSLRLSCAASEDTYCIYDMGWFRQAPGREREGVAAIDIDGSARYAASVEGRFTISQDNAANTLYLQMNSLKPEDTAMYFCAAR  NAADGLCDGGDCADFAYWGQGTQVTVSS
```

Appendix Figure S12. The sequencing and alignment analysis of Nbs 1G9 and 1D8.

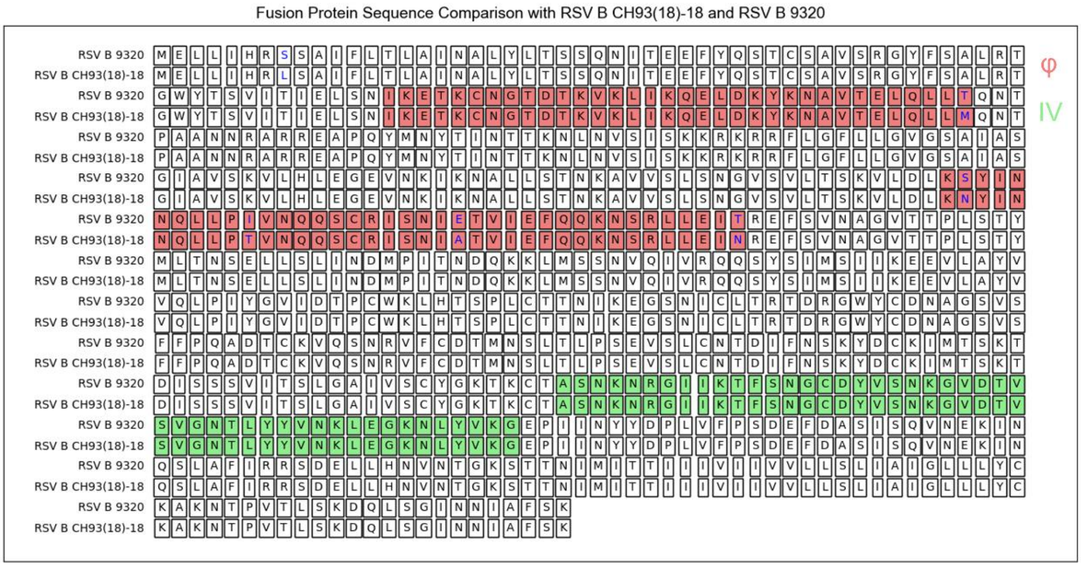

Appendix Figure S13. Genetic sequencing and alignment analysis of F gene in RSVB [CH93(18)-18] and RSV B9320. The F protein sequence of RSV B9320 was downloaded from GenBank (GenBank: AY353550.1), and the full-length sequence of RSVB [CH93(18)-18] was obtain by DNA sequence (Sangon Biotech (Shanghai) Co., Ltd.). We performed sequence alignment analysis by Python package and represented as color-coded boxes. The red background represents site Ø, the green background represents site IV, mutant amino acid in blue.

Appendix Table S1. Cryo-EM data collection, refinement and validation

	#1 RSV A2 DS Cav1:1G9 (EMD-63216) (PDB: 9LM5)	#2 RSV A2 DS Cav1:1D8 (EMD-63217) (PDB: 9LM6)
**Data collection and processing**		
Magnification	50,000	50,000
Voltage (keV)	300	300
Pixel size (Å)	0.95	0.95
Defocus	1.0-2.0	1.0-2.0
Symmetry imposed	C3	C3
Map Resolution (Å)	2.77	3.26
FSC threshold	0.143	0.143
Map sharpening B factor (Å2)	-131.8	-197
**Model composition**		
Non-hydrogen atoms	13,479	13,350
Protein residues	1,755	1,731
Water	0	0
**RMS deviation from ideality**		
Bond lengths (Å)	0.005	0.005
Bond angles (º)	0.979	1.041
**Validation**		
MolProbity score	2.10	2.90
Sidechain outliers	4.70	13.00
Clash score	10.00	14.00
Rotamer outliers (%)	2.49	11.79
**Ramachandran statistics**		
Favored regions (%)	96.03	93.29
Allowed regions (%)	3.97	6.71
Outlier (%)	0	0

Appendix Table S2. Binding site of nanobodies on RSV F and sequence conservation

The frequency of conserved amino acid residues in the F protein of RSV A and B viruses within the nanobody binding site					
		RSV A (N = 5535)		RSV B (N = 4343)	
**Location in F subunit**	**Amino acid position in F protein**	**Consensus residue**	**Conservation rate (%)**	**Consensus residue**	**Conservation rate (%)**
F2	429	R	100	R	100
	430	G	100	G	100
	431	I	100	I	100
	432	I	99.91	I	99.98
	433	K	100	K	99.95
	*				
	443	S	99.96	S	99.98
	444	N	100	N	100
	445	K	99.96	K	99.98
	446	G	99.98	G	99.98
	*				
	465	K	99.91	K	99.95
	466	S	98.93	N	99.86
	467	L	99.73	L	99.82
	468	Y	100	Y	99.98
	469	V	99.98	V	99.93
	470	K	99.96	K	99.98
