# Supplementary figures and images for: Broadly neutralizing nanobodies target a defined structural pivot site on the RSV fusion protein
Source: EMBO Mol Med. 2026 Apr 7;18(5):1866–83. doi: 10.1038/s44321-026-00412-w (PMC13179361; doi:10.1038/s44321-026-00412-w)

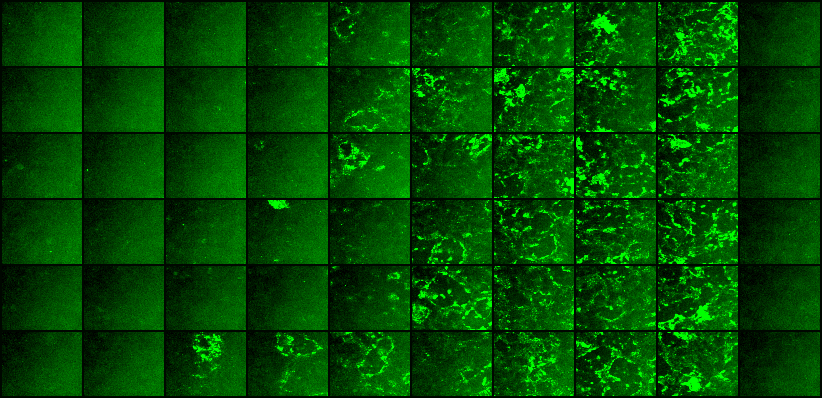

Supplement: Supplementary file 5 — Source data Fig. 3 [file 44321_2026_412_MOESM5_ESM.zip › Source Data-Figure 3/3A/RSV A2-neutralization-1G9-1D8-Fc/HTS - FITC-1.tif]

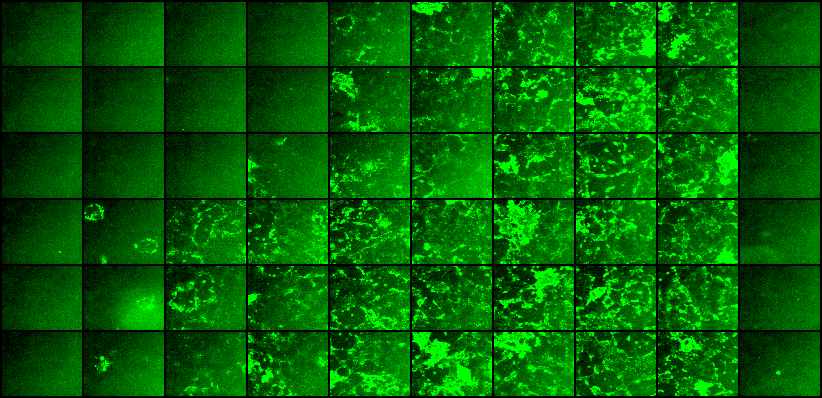

Supplement: Supplementary file 5 — Source data Fig. 3 [file 44321_2026_412_MOESM5_ESM.zip › Source Data-Figure 3/3A/RSV A2-neutralization-1G9-1D8-his/HTS - FITC-1.tif]

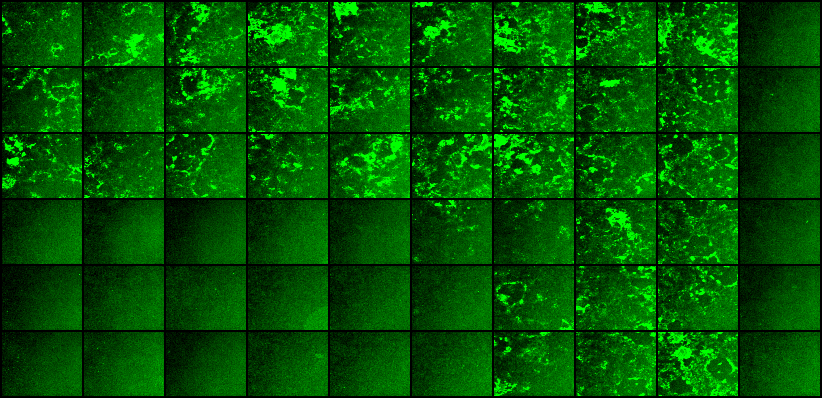

Supplement: Supplementary file 5 — Source data Fig. 3 [file 44321_2026_412_MOESM5_ESM.zip › Source Data-Figure 3/3A/RSV A2-neutralization-PVZ-nirsevimab-Fc/HTS - FITC-1.tif]

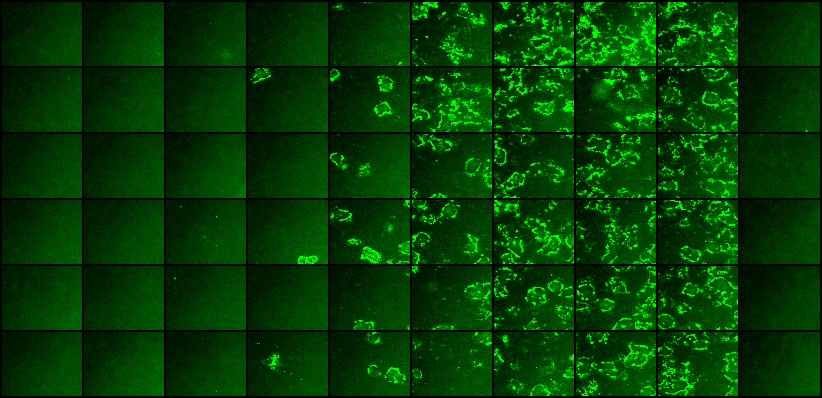

Supplement: Supplementary file 5 — Source data Fig. 3 [file 44321_2026_412_MOESM5_ESM.zip › Source Data-Figure 3/3B/RSV B-neutralization-1G9-1D8-Fc/HTS - FITC-1.tif]

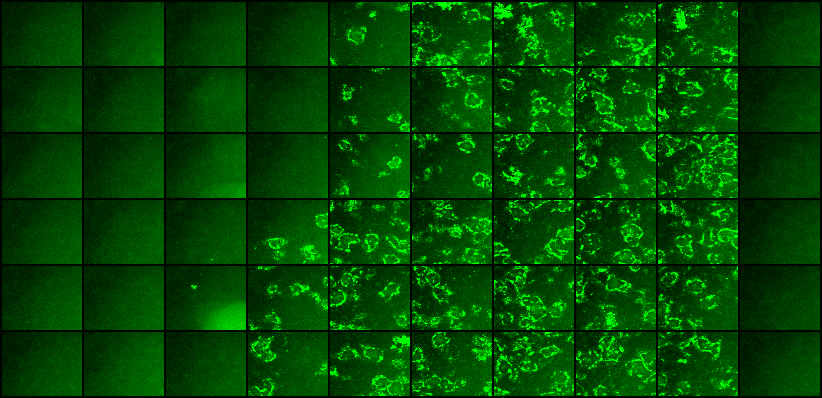

Supplement: Supplementary file 5 — Source data Fig. 3 [file 44321_2026_412_MOESM5_ESM.zip › Source Data-Figure 3/3B/RSV B-neutralization-1G9-1D8-His/HTS - FITC-1.tif]

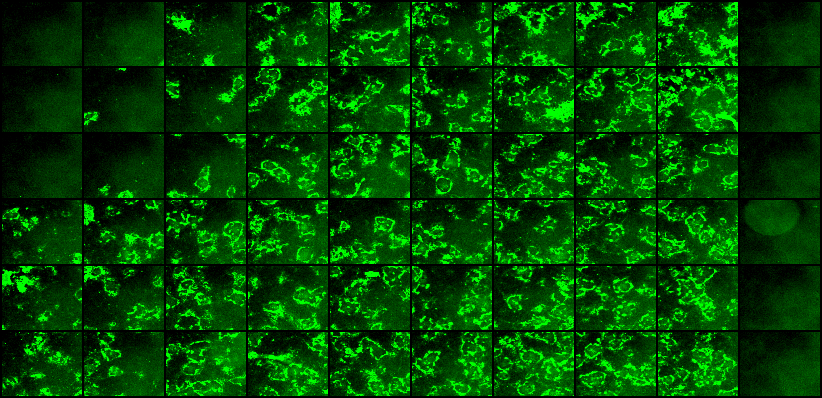

Supplement: Supplementary file 5 — Source data Fig. 3 [file 44321_2026_412_MOESM5_ESM.zip › Source Data-Figure 3/3B/RSV B-neutralization-PVZ-nirsevimab-Fc/HTS - FITC-1.tif]

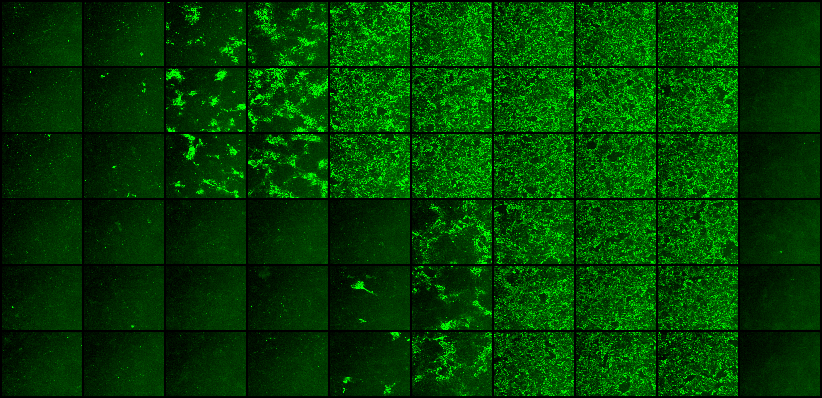

Supplement: Supplementary file 5 — Source data Fig. 3 [file 44321_2026_412_MOESM5_ESM.zip › Source Data-Figure 3/3C/RSV Long-neutralization PVZ-nirsevimab-Fc-100nM/HTS - FITC-1.tif]

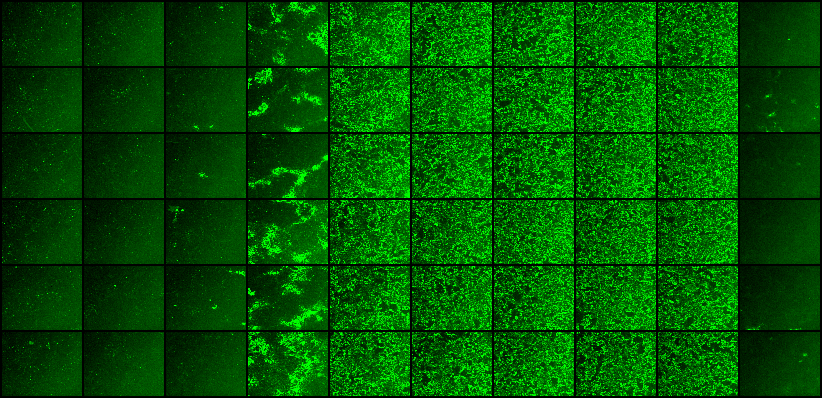

Supplement: Supplementary file 5 — Source data Fig. 3 [file 44321_2026_412_MOESM5_ESM.zip › Source Data-Figure 3/3C/RSV Long-neutralization1G9-1D8-Fc--100nM/HTS - FITC-1.tif]

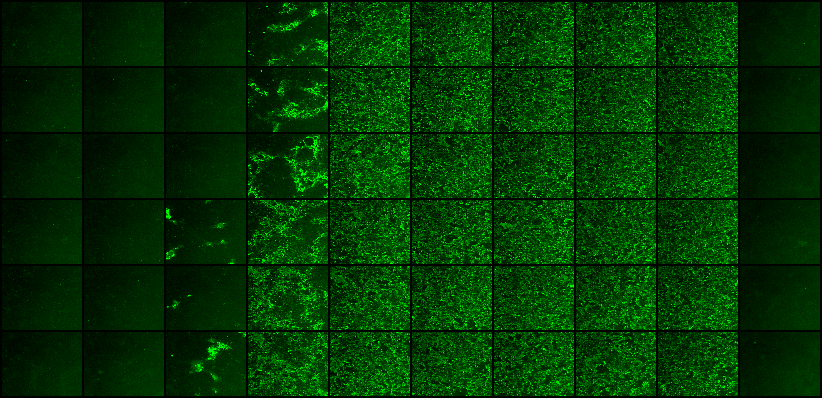

Supplement: Supplementary file 5 — Source data Fig. 3 [file 44321_2026_412_MOESM5_ESM.zip › Source Data-Figure 3/3C/RSV Long-neutralization1G9-1D8-His-100nM/HTS - FITC-1.tif]

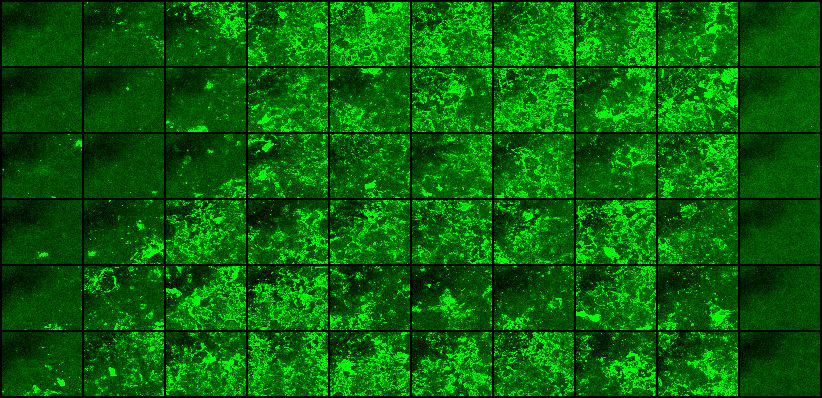

Supplement: Supplementary file 5 — Source data Fig. 3 [file 44321_2026_412_MOESM5_ESM.zip › Source Data-Figure 3/3D/RSV A2-fusion inhibition -1G9-1D8-Fc/HTS - FITC-1.tif]

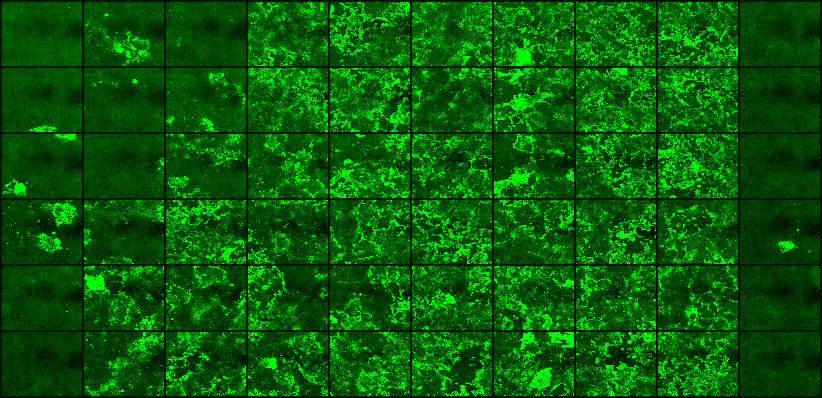

Supplement: Supplementary file 5 — Source data Fig. 3 [file 44321_2026_412_MOESM5_ESM.zip › Source Data-Figure 3/3D/RSV A2-fusion inhibition -1G9-1D8-His/HTS - FITC-1.tif]

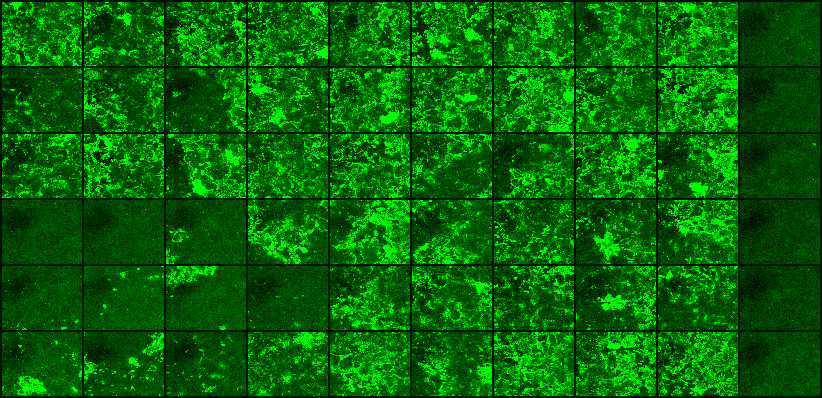

Supplement: Supplementary file 5 — Source data Fig. 3 [file 44321_2026_412_MOESM5_ESM.zip › Source Data-Figure 3/3D/RSV A2-fusion inhibition -PVZ-nirsevimab-Fc/HTS - FITC-1.tif]

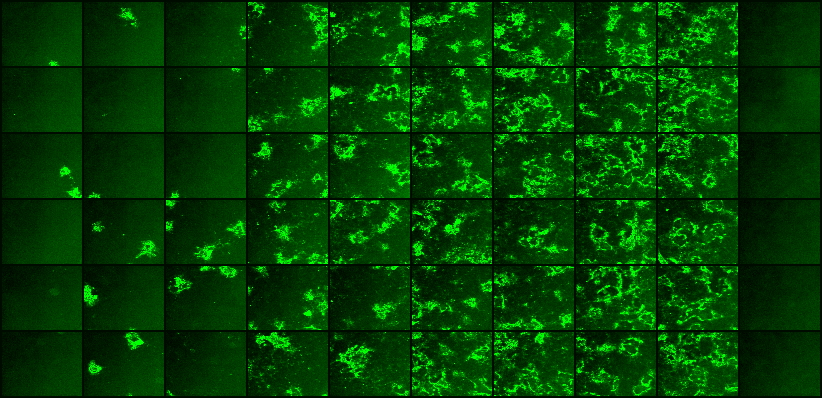

Supplement: Supplementary file 5 — Source data Fig. 3 [file 44321_2026_412_MOESM5_ESM.zip › Source Data-Figure 3/3E/RSV B-Fusion inhibition-1G9-1D8-Fc/HTS - FITC-1.tif]

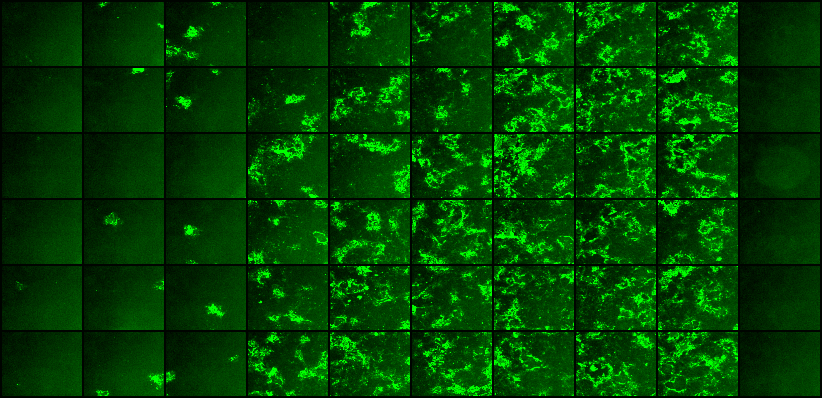

Supplement: Supplementary file 5 — Source data Fig. 3 [file 44321_2026_412_MOESM5_ESM.zip › Source Data-Figure 3/3E/RSV B-Fusion inhibition-1G9-1D8-His/HTS - FITC-1.tif]

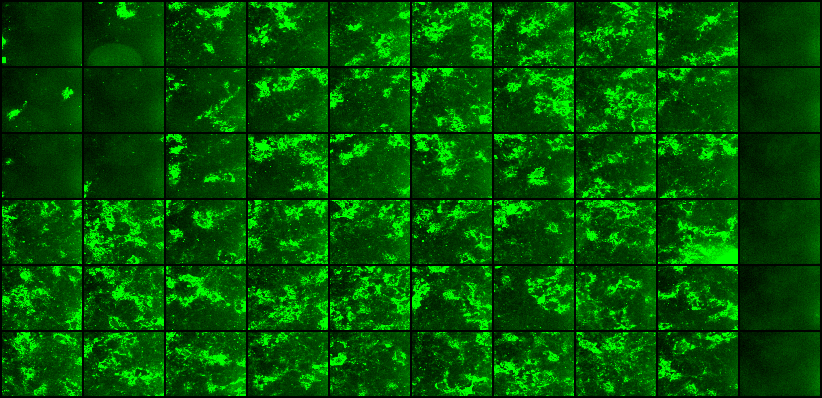

Supplement: Supplementary file 5 — Source data Fig. 3 [file 44321_2026_412_MOESM5_ESM.zip › Source Data-Figure 3/3E/RSV B-Fusion inhibition-PVZ-8897-Fc/HTS - FITC-1.tif]

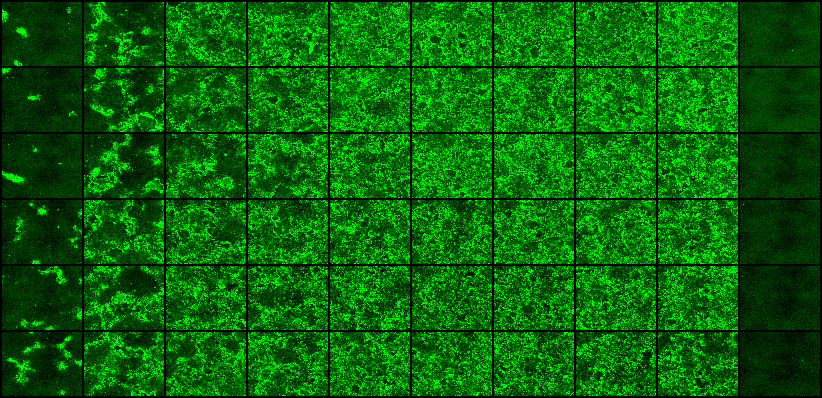

Supplement: Supplementary file 5 — Source data Fig. 3 [file 44321_2026_412_MOESM5_ESM.zip › Source Data-Figure 3/3F/RSV Long-fusion inhibition-1G9-1D8-Fc/HTS - FITC-1.tif]

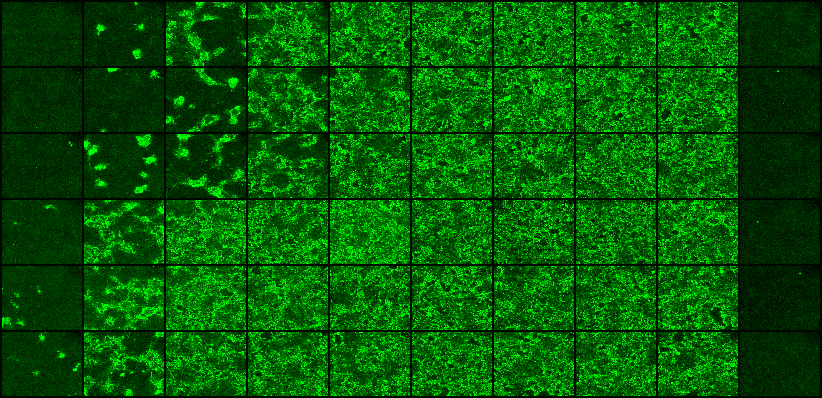

Supplement: Supplementary file 5 — Source data Fig. 3 [file 44321_2026_412_MOESM5_ESM.zip › Source Data-Figure 3/3F/RSV Long-fusion inhibition-1G9-1D8-his/HTS - FITC-1.tif]

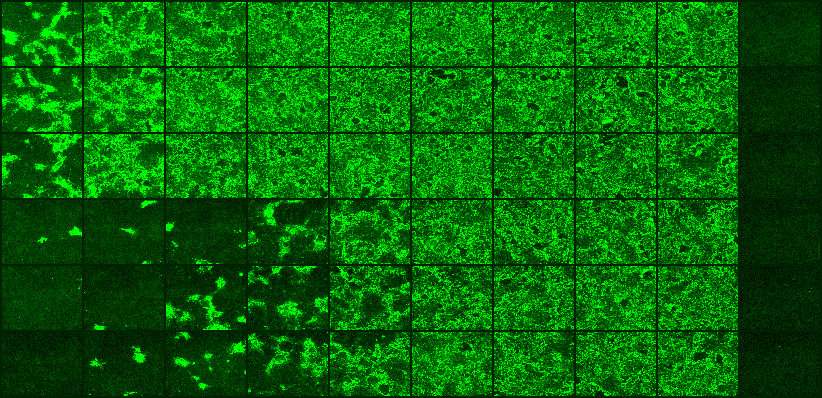

Supplement: Supplementary file 5 — Source data Fig. 3 [file 44321_2026_412_MOESM5_ESM.zip › Source Data-Figure 3/3F/RSV Long-fusion inhibition-PVZ-nirsevimab-Fc/HTS - FITC-1.tif]

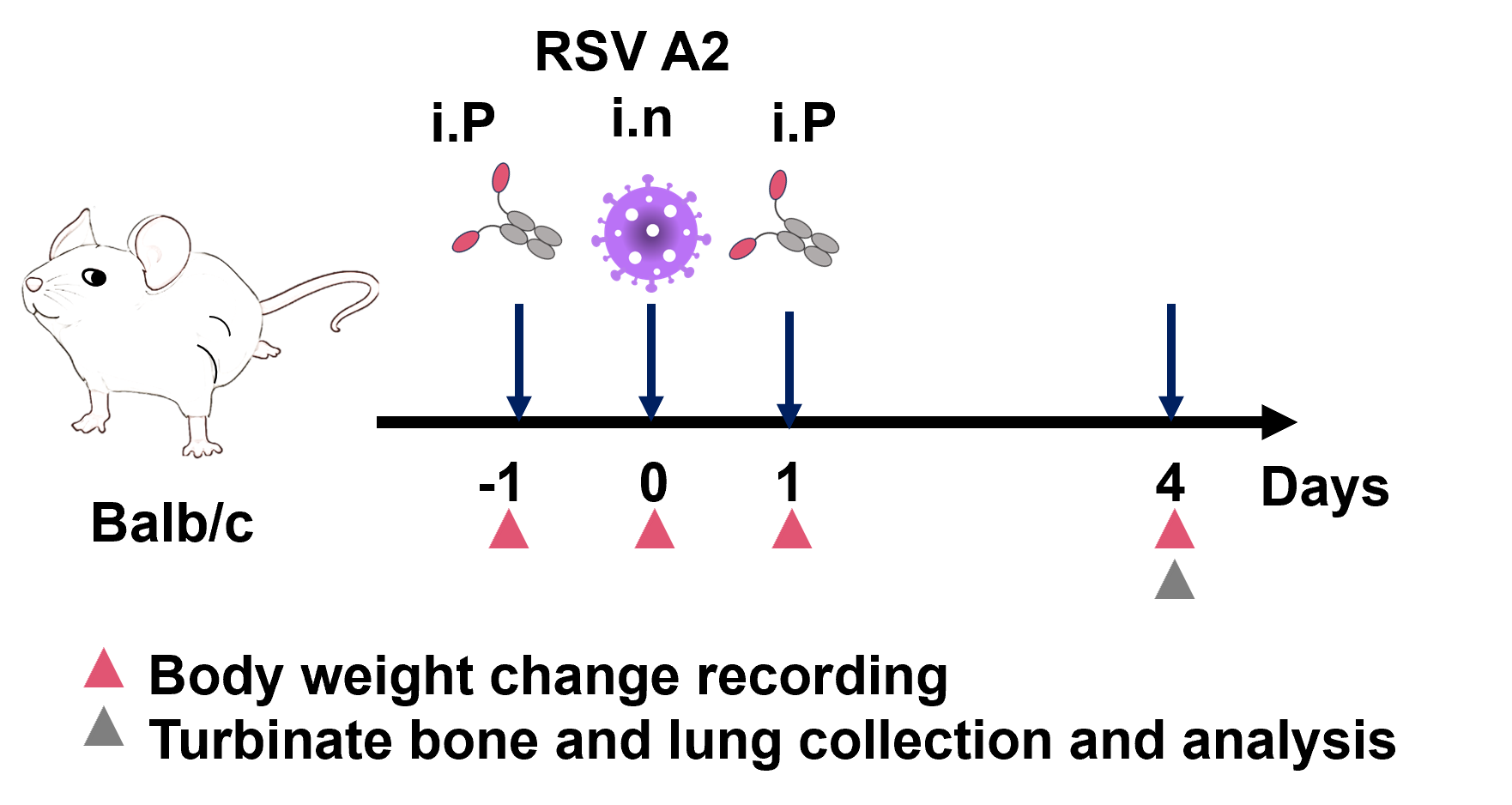

Supplement: Supplementary file 9 — Source data Fig. 7 [file 44321_2026_412_MOESM9_ESM.zip › Source Data-Figure 7/7A/animal experiments.png]
